# Complete mitochondrial genome of the juvenile *Gymnodiptychus dybowskii* (Cypriniformes, Cyprinidae, Schizothoracinae) from Ili River by high-throughput sequencing and the phylogenetic relationship of Schizothoracinae species

**DOI:** 10.1080/23802359.2019.1704663

**Published:** 2020-01-10

**Authors:** Jiangong Niu, Tao Zhang, Hong Liu, Jiangwei Hu, Renming Zhang, Hui Zhang

**Affiliations:** a Xinjiang Fishery Research Institute, Urumqi, China;; b Laboratory for Marine Ecology and Environmental Science, Qingdao National Laboratory for Marine Science and Technology, Qingdao, China;; c CAS Key Laboratory of Marine Ecology and Environmental Sciences, Institute of Oceanology, Chinese Academy of Sciences, Qingdao, China;; d Center for Ocean Mega‐Science, Chinese Academy of Sciences, Qingdao, China

**Keywords:** *Gymnodiptychus dybowskii*, juvenile fish, mitochondrial genome, high-throughput sequencing

## Abstract

The complete mitochondrial genome of the juvenile *Gymnodiptychus dybowskii* collected from Ili River was determined by high-throughput sequencing. The mitogenome is a circular molecule 16,657bp in length, including 13 protein-coding genes, 2 ribosomal RNA genes, 22 transfer RNA genes, and a control region. The TAS, central CSB and CSB were detected in the control region. The gene contents of the mitogenome are identical to those observed in most bony fishes. The NJ phylogenetic tree showed that *G. dybowskii* clustered into one branch with the species from the same genus.

High-throughput sequencing has revolutionized the field of molecular biology through the rapid and cost effective collection of large amounts of genomic data (Schuster 2008). It could provide an effective platform for the development of the mitochondrial genome that can be used to provide insight into population processes and the evolutionary history of species. By exploiting certain tissue types, such as muscle, total genomic DNA extractions can contain high concentrations of mitochondrial DNA which may then be overrepresented in high-throughput sequencing analyses (Dalziel et al. 2005).

*Gymnodiptychus dybowskii* distributes only in some waters of Xinjiang, such as Ili River and Kaidu River (Niu et al. 2015). It is a representative indigenous and ecologically important species in Xinjiang aquatic ecosystems (Cai et al. 2017). However, there is no genetic study on its juvenile stage. In this study, we use high-throughput sequencing by Illumina Hiseq analysis to determine the mitogenome of the juvenile *G. dybowskii* collected from Ili River (43°49.811′N, 82°24.277′E) in April 2019. The specimen is preserved in the fish herbarium of Xinjiang Fishery Research Institute with the No. XLC-2019-04-06.

The complete mitogenome of *G. dybowskii* was 16,657 bp in length (GenBank accession no. MN422094), with the nucleotide composition as A (26.87%), T (28.33%), G (26.18%), and C (18.62%). As in other vertebrates (Miya et al. 2001), it contained 13 protein-coding genes, 2 rRNA genes (12S rRNA and 16S rRNA), 22 tRNA genes, and a control region. Most mitochondrial genes of *G. dybowskii* were encoded on the H-strand, with only ND6 and eight tRNA (Gln, Ala, Asn, Cys, Tyr, Ser-UCN, Glu, and Pro) genes encoded on the L-strand. Among 13 protein coding genes, two overlapping reading frames were detected on the same strand. The ATPase 6 and ATPase 8 overlap by six nucleotides, and ND4 and ND4L share six nucleotides. ND5 and ND6 overlap by three nucleotides on the opposite strand. ATG is the initiation codon of all protein coding genes. TAA is the stop codon for six genes (ND6, COI, ATPase 6, COIII, ND4L, and ND5), the other genes have incomplete stop codons TA or T–, which are presumably completed as TAA by post-transcriptional polyadenylation (Ojala et al. 1981). The 12S and 16S ribosomal RNA genes of *G. dybowskii* comprise 968 bp and 1663 bp, respectively. They are located between tRNA^Phe^ and tRNA^Leu^ (UUR) as they are in other vertebrates (Zhang and Xian 2018). The 22 tRNA genes are interspersed in the genome and range in size from 65 to 72 bp and fold into cloverleaf secondary structures with normal base paring. The control region of *G. dybowskii* is located between tRNA^Pro^ and tRNA^Phe^, and was determined to be 1160 bp in length. The TAS, central CSB, and CSB were detected in the control region, which is similar to most bony fishes (Zhang et al. 2013). Phylogenetic relationship was revealed by NJ tree among 15 Schizothoracinae species based on complete mitogenome. The NJ phylogenetic tree showed that *G. dybowskii* clustered into one branch with the species from the same genus (Figure 1).

**Figure 1.**
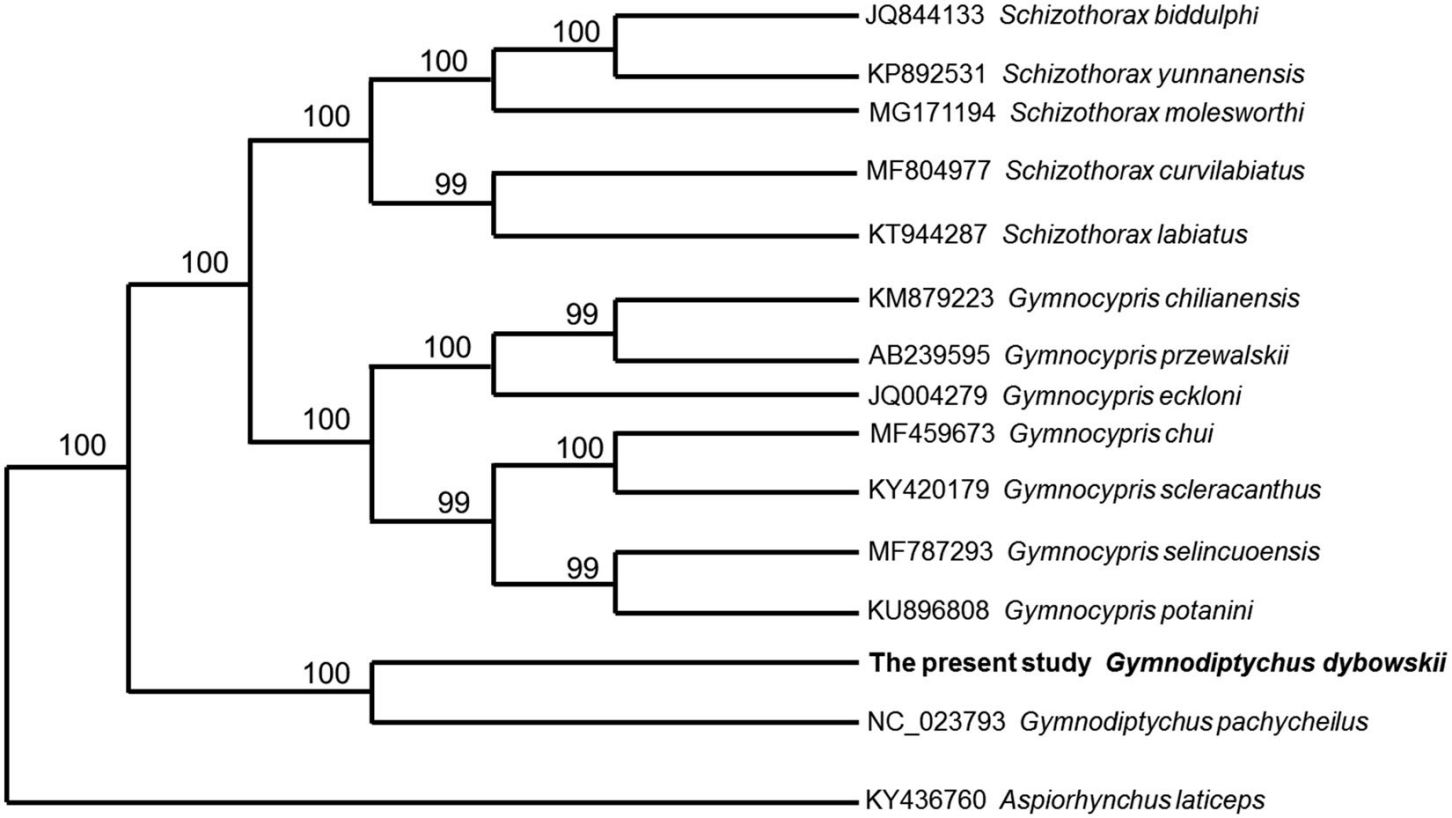
Phylogenetic relationship revealed by NJ tree among 15 Schizothoracinae species.
